# Phenomenal Diversity of the Photosynthetic Apparatus Evolved in Aerobic Anoxygenic Phototrophs

**DOI:** 10.3390/microorganisms13112446

**Published:** 2025-10-25

**Authors:** Vladimir Yurkov, Katia Messner

**Affiliations:** Department of Microbiology, University of Manitoba, Winnipeg, MB R3T 2N2, Canada; messner1@myumanitoba.ca

**Keywords:** aerobic anoxygenic phototrophs, bacterial photosynthesis, bacteriochlorophyll, light-harvesting complexes, reaction center, carotenoids, photosynthetic gene cluster

## Abstract

Aerobic anoxygenic phototrophs (AAPs) are intrinsically paradoxical; these species use a pathway commonly found in oxygen-deprived environments called anoxygenic photosynthesis, as a supplementary energy source to their obligately aerobic respiration. At the surface, such a combination seems odd, but AAPs thrive in a plethora of environments and are phylogenetically broad, suggesting that this feature is advantageous and ecologically valuable. The range of habitats and taxonomy have been reviewed, yet the main element which unites the group, their anoxygenic photosynthesis, which is diverse in its components, has not received the deserved attention. The intricate light-capturing photosynthetic complex forms the site of photon-induced energy transfer and therefore, the core basis of the process. It has two parts: the reaction center and light harvesting complex(es). The variability in composition and overall usage of the apparatus is also reflected in the genome, specifically the photosynthetic gene cluster. In this review, what is known about the differences in structure, light wavelength absorption range, activity, and related genomic content and the insights into potential AAP evolution from anaerobic anoxygenic phototrophs will be discussed. The work provides an elegant summation of knowledge accumulated about the photosynthetic apparatus and prospects that can fill yet remaining gaps.

## 1. Introduction

**Figure 1:**
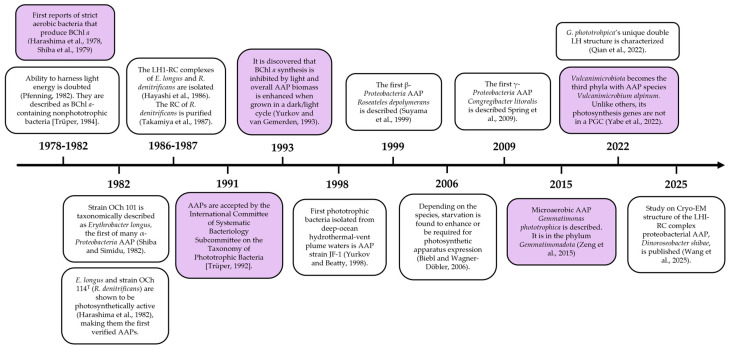
Timeline of major AAP discoveries. Purple indicates the most impactful revelations [3,4,5,7,8,11,12,13,17,23,24,25,26,27,28,29,30,31].

Anoxygenic photosynthesis is an ancient free energy-producing process that formed the foundation of life on Earth prior to its oxygenation [1]. It is a cyclic electron (e^−^) transport chain powered by light, which generates a proton gradient across the membrane that stimulates ATP synthesis. Depending on the bacterium, a variety of inorganic (e.g., H_2_S and H_2_) and partially reduced organic e^−^ donors may be involved, and through reverse e^−^ flow, a set of e^−^ carriers for other metabolic pathways may be generated (e.g., NADH). As the name suggests, no oxygen is produced. For this reason, since its initial discovery in the late 1800s, it was presumed it could only take place under anaerobic conditions [2]. All bacteria isolated, purified, and studied that were capable of harnessing light-energy through anoxygenic photosynthesis were only able to use it when oxygen was absent. That was until publications describing strain OCh101 (*Erythrobacter longus*) in 1978 and OCh114 (*Roseobacter denitrificans*) in 1979, as they are strict aerobes that synthesize photosynthetic pigment bacteriochlorophyll *a* (BChl *a*) in the dark and in the presence of oxygen [3,4,5,6]. These photoheterotrophs redefined what was stereotypically thought about this process and became the first representatives of a group called aerobic anoxygenic phototrophs (AAPs), which utilize solar energy as a supplemental source for respiration. However, in their initial discovery, there were doubts that they could photosynthesize due to implications of insufficient evidence and, thus, were not classified as such [7,8]. Shortly after, released articles provided undoubtable proof for photosynthesis under aerobic conditions [9,10,11,12]. Hence, they were finally accepted as AAPs (Figure 1) [13]. Since then, many more have been recovered from a variety of environments: freshwater and saline lakes [14], freshwater sulfide hot springs [15,16], hydrothermal black smoker vents [17], hypersaline springs [18], toxic gold mine drainage systems [19], and biological soil crusts [20]. Furthermore, they have been found to comprise > 10% [21] of total microbial species in the largest ecosystem in the world, the ocean [22], showing that the group is globally distributed and has an important niche in the biosphere [22].

The key uniting feature of the group is the requirement of oxygen for respiration and anoxygenic photosynthesis [2]. They cannot grow exclusively using the energy acquired from light. Instead, it serves as a supplemental source to their otherwise chemoheterotrophic metabolism. In fact, no true AAP can grow autotrophically by fixing CO_2_ or synthesizing ribulose-1,5-bisphosphate carboxylase/oxygenase (RuBisCO), a key enzyme in carbon fixation via the Calvin Cycle [32]. Therefore, AAP photosynthesis is inherently different from other anoxygenic phototrophs despite using a quite similar system. As such, they form a distinct physiological group of photoheterotrophic bacteria.

**Figure 2:**
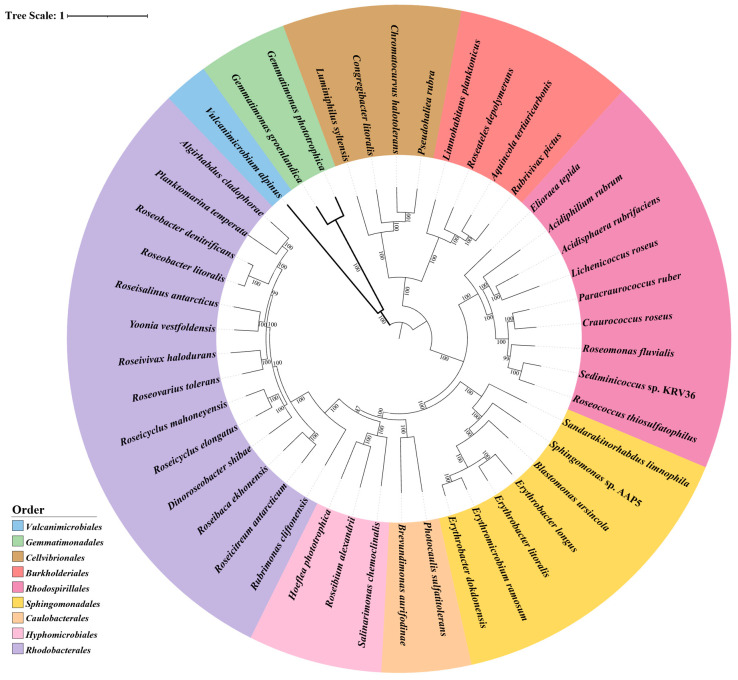
Phylogenetic tree of multi-loci sequence alignment of 457 genes from PGFams identified within the selected genomes of AAPs with proven photosynthetic apparatus expression. Bold branches indicate AAPs outside of *Pseudomonadota*. Tree generated on BV-BRC [41] used mafft as the alignment method and LG as the substitution model, and the branch support values were generated from 100 rounds of ‘Rapid bootstrapping’ in RaxML (v8.2.12) and visualized with iTOL [42]. The tree scale is defined as the mean number of substitutions per site, averaging both nucleotide and amino acid changes. GenBank assembly accession numbers are included in Supplementary Table S1.

The majority of known AAP fall within the α-, β-, and γ-*Proteobacteria* classes. In some cases, methylotrophic bacteria and photosynthetic rhizobia have been classified as AAPs and are discussed extensively in other works [9,33,34,35], and we only briefly mention them where appropriate. However, members that fit the broadest definition of the group have included species from *Gemmatimonadota* [28], *Acidobacteriota* [36], *Vulcanimicrobiota* [30], and, based on metagenomic sequencing, possibly *Myxococcota* (formerly classified as δ-*Proteobacteria*) [37,38] phyla. This makes AAPs the most phylogenetically extensive group of phototrophic bacteria (Figure 2). However, the nature of their photosynthesis is the least understood and studied of the classified anoxygenic phototrophs. Work in the last few decades has moved towards closing the gap of knowledge on the light-harnessing capabilities and provided substantial evidence for its value in survival, dispelling previous doubts [39,40]. Another aspect of AAP diverseness is in the structure and expression of the photosynthetic apparatus. Although very important and interesting, it has had quite limited discussion; therefore, our review will explore this exciting facet of AAP. Here, the variation in pigment–protein complex structures, photosynthetic gene cluster (PGC) composition, arrangement, and regulation, as well as environmental factors that affect how they employ photosynthesis, will be analyzed to highlight the remarkable diversity found within the group.

## 2. The Aerobic Anoxygenic Photosynthesis Pathway

**Figure 3:**
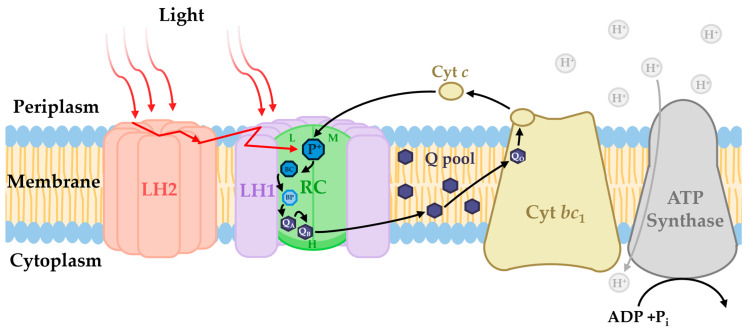
Photosynthesis pathway in AAPs. Lines indicate movement of *hυ* (red), e^−^ (black), and protons (grey). Abbreviations: Q, quinone; BC, bacteriochlorophyll; BP, bacteriopheophytin; cyt, cytochrome; LH, light-harvesting complex; RC, reaction center.

Despite the fundamentally different conditions where AAP use photosynthesis, the cyclic pathway is very similar to that of purple non-sulfur bacteria (PNSB); however, due to the redox potential of the e^−^ carriers, photoinduced e^−^ transfer is operative only under aerobiosis [2]. First, light energy at varying wavelengths is captured by pigments in the light-harvesting (LH) complex(es) and funneled to the reaction center (RC). There, the photons excite a pair of BChl *a* (P), which become photooxidized as a result (P^+^), with e^−^ passing to an auxiliary BChl, bacteriopheophytin (BPhe), then to the ubiquinone primary e^−^ acceptor located at the Q_A_ site. Here, a fundamental difference lies between AAPs and other anoxygenic phototrophs. AAPs’ Q_A_ binding site has a higher midpoint potential, which optimizes the process for aerobic conditions and renders it useless when oxygen is absent, where it would be quickly over reduced and unable to transfer e^−^ (Section 3.1 and Section 3.6). The e^−^ are then passed to a secondary ubiquinone located at Q_B_, which exits the RC and moves through the membrane to the cytochrome (cyt) *bc*_1_ complex. At the Q_O_ site, the ubiquinone is oxidized and the e^−^ transferred to cyt *c*_2_, which returns to the RC via the periplasmic space (Figure 3) [2]. Through the process, an electrochemical potential is generated across the membrane via the transfer of protons from the cytoplasm to the periplasm via the Q cycle [43]. The proton gradient is utilized by the cell to produce chemical energy in the form of ATP via photophosphorylation (Figure 3). As such, photosynthesis is an alternate source of energy to aerobic respiration, allowing AAP to spare some organic compounds for anabolic activities or for essential tasks under nutrient-limited conditions [44]. Below, the current knowledge of the crucial components that comprise the photosynthetic apparatus, their extensive diversity, and the genes involved are discussed.

## 3. The Photosynthetic Apparatus: Typical and Atypical

Photosynthesis requires the precise coordination of many intricate factors, pigments, and proteins to ultimately turn photons into chemical energy via the production of ATP. The process comprises the photosynthetic complex (also known as the pigment–protein complex), which consists of the RC, LH1, and in some cases, an LH2, the cyt *bc*_1_ complex, and e^−^ transporters, which carry e^−^ to and from the RC. In this section, the different parts of the photosynthetic apparatus will be defined, highlighting the variation that exists among AAPs.

### 3.1. Reaction Center (RC)

**Figure 4:**
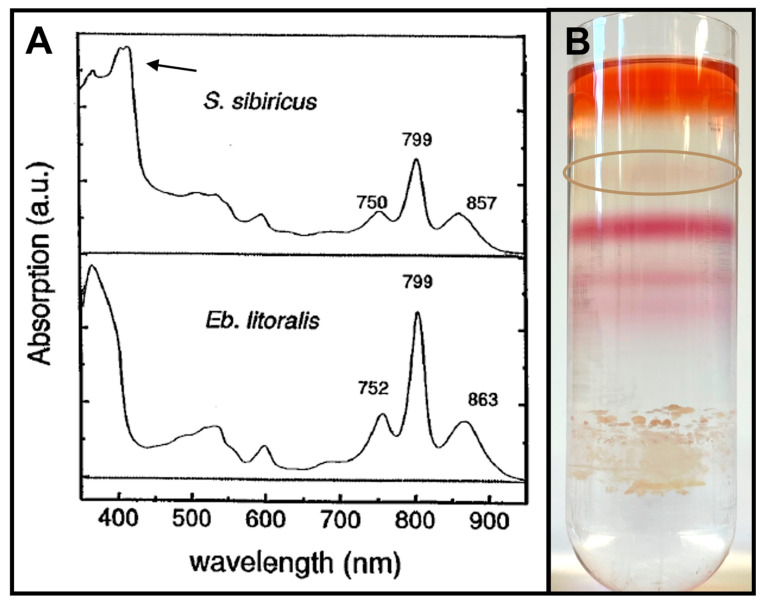
The two different types of RC isolated from AAPs. (**A**) The RC of *Sandaracinobacter sibiricus* containing bound cyt *c* subunit (arrow) and *Erythrobacter litoralis*, which is cyt free [45]. (**B**) A sucrose density gradient (0.3, 0.6, 0.9, and 1.2 M) loaded with AAP strain CK155 membranes showing the RC (beige circle), free pigments on top (red), and LH1-RC bands (purple). Isolated membranes were treated with 0.5% LDAO and mixed for 30 min at room temperature before being loaded on a gradient and spun overnight at 32,000 rpm for 16 h.

The RC is the central, most important unit required for photosynthesis. It captures light energy that is inevitably used to generate chemical energy in the form of ATP. Easing initial doubts, the RC in AAPs was proven to be operational multiple times in some of the earliest known members [11] and was then first purified from *Rb. denitrificans* [23]. Followed by isolation from many other marine and freshwater species [45], the mode of anoxygenic photosynthesis in the presence of oxygen was validated. AAP RCs have a similar composition to PNSB: 4 BChl *a* molecules, 2 BPhe, 2 quinones, non-heme iron, and a photosynthetic carotenoid all organized within 3–4 proteins named H, L, and M and the optional cyt *c* subunit (named after the cyt, which may be bound and act as an intermediary e^−^ donor to P^+^) [2,46]. In absorption spectra of purified RC, there are three major peaks: ~750 nm, due to BPhe, ~800 and ~860 nm, indicating BChl *a* [2]. Among AAPs, an RC bound cyt *c* subunit is an inconsistent component. In *E. longus*, *E. litoralis*, *Erythromicrobium ramosum*, *Er. hydrolyticum*, *Er. ezovicum*, and *Porphyrobacter meromictius*, there is no RC-bound cyt (Figure 4) (genus abbreviations are provided in Supplementary Table S2) [45,47,48,49]. In contrast, *Blastomonas ursincola*, *Sandarcinobacter sibiricus*, *Roseococcus thiosulfatophilus*, *Rb. denitrificans*, *Roseicyclus mahoneyensis*, and *Chromatocurvus halotolerans* contain cyt *c* bound to the RC (Figure 4) [45,47,48,50]. Having the cyt *c* directly associated with the RC allows the P^+^ pair to be re-reduced at a quicker rate than those with only a soluble cyt *c* [48]. The cyt *c* subunits found in AAP and anaerobic anoxygenic photoautotrophs vary at the N-terminus and how they associate with the membrane. The latter contains a Cys residue, which is used for a lipid anchor attachment post-translation while AAPs rely exclusively on the transmembrane N-terminal helix for adherence [51,52]. Most cyt *c* subunits comprise 4 hemes to facilitate the transfer of e^−^ to the P^+^ pair, but *D. shibae* only has 3, with modeled interactions proposing that cytochrome *c*_2_ donates the e^−^ to the heme closest to P^+^ [31]. Since the other distant hemes are likely not being used, the tri-heme version may represent a transitional structure between anoxygenic phototrophs with and without a cyt *c* subunit [31].

As mentioned above, binding site Q_A_ in AAPs has a high midpoint potential, while in anaerobic anoxygenic phototrophs, it is significantly lower, so that Q_A_ can accept e^−^ under reducing conditions (Table 1). Therefore, AAPs with a higher Q_A_ midpoint potential will have their anoxygenic photosynthesis pathway halted under anaerobic conditions because the ubiquinone at that site will already be reduced and unable to accept electrons from BPhe [53]. Even in AAPs with a lower Q_A_ binding site midpoint potential, oxygen is still required. This is because it relies on its aerobic respiratory pathway to manage the redox state of the quinone pool and prevent non-specific quinone overreduction, since they do not have alternative mechanisms [54]. Such differences form the key to variability of function but only scratch the surface of the phenotypic heterogeneity in the AAP photosynthetic complex. Another fundamental component, the LH complex(es), have a wide range of absorbance spectrum diversity in the group.

### 3.2. Light-Harvesting Complex 1 (LH1)

**Figure 5:**
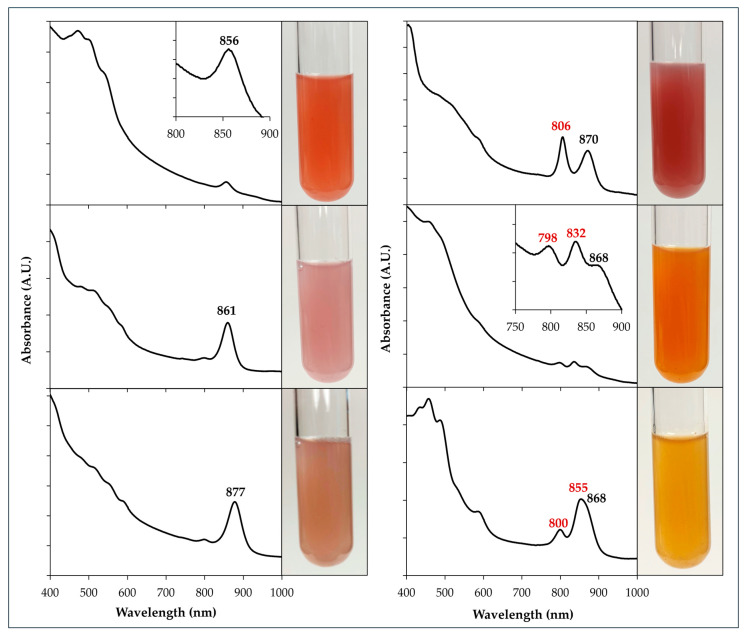
The color and whole cell absorbance spectra of various AAPs. Values indicate absorption peaks of BChl *a* incorporated in LH1 (black) and/or LH2 (red). From left to right: top row (*Roseococcus thiosulfatophilus*, *Roseicyclus mahoneyensis*), middle row (*Photocaulis rubescens*, *Erythromicrobium ramosum*) and bottom row (*Chromatocurvus halotolerans*, *Polymorphobacter* sp. FW250). Absorption spectra of whole cells were prepared and measured as described [48].

LH1 is an antenna that collects and shuttles light energy acquired from associated pigments to the RC ensuring a constant supply for photosynthesis. The complex usually contains 16 individual units (sometimes 17, as in *D. shibae* [31]), each comprising αβ dimer subunits, BChl, and carotenoids [46,57]. Due to the number of BChl molecules in LH1 greatly surpassing what is found in the RC (32 vs. the standard 4) [58], the LH1 peak (usually ~870 nm) tends to overlap the ~860 nm peak of the RC in the whole cell absorbance spectrum. Interestingly, there are a variety of atypical LH1 complexes that deviate from this standard (Table 2, Figure 5). For example, *Rc. thiosulfatophilus*, *Paracraurococcus ruber*, and strain CK155 have an LH1 with an 856 or 854 nm peak, shifting closer to the typical LH2 peaks (Section 3.3). Slightly less blue-shifted are the LH1 peaks discovered in *Photocaulis rubescens* (861 nm), *Ph. sulfatitotolerans* (860 nm), and *E. dokdonensis* (862 nm). Conversely, there are AAPs with more red-shifted LH1 found throughout the *Pseudomonadota* (formerly *Proteobacteria*): from α-(*Roseovarius tolerans*, 878 nm; *R. elongatum*, 879 nm), β-(*Roseateles depolymerans*, 873 nm) and γ-*Proteobacteria* (*C. halotolerans*, 877 nm; *Congregibacter litoralis*, 874 nm). The shifts in wavelengths of light absorbed are a result of how BChl is incorporated into the structure, which is affected by changes in the amino acid sequence. In certain variants of peripheral LH complexes, such as LH4 (Section 3.4.2), AAPs with identical sequences at key sites on the α-subunit share very similar spectral features [59]. This likely applies to LH1 as well.

In structurally characterized LH1-RC units, the LH1 complex appears to completely encircle the RC [29,31,46,64]. However, in some PNSB, there is a channel, presumably to ease exit of the quinone, so it can move to the cyt *bc*_1_ complex [57]. However, none of the very few AAP LH1-RC structures characterized via cryo-electron microscopy (cryo-EM) have this [29,31,46,64]. Therefore, it is uncertain whether such openings in the assembled LH1 complex occur in the group and/or if that feature is species-dependent. Comparative analysis of αβ subunit’s amino acid sequences could enhance our understanding of protein–pigment interactions and how they result in the absorbance spectral shifts seen in various AAPs. Furthermore, if such aspects vary from their anaerobic counterparts can be explored. In *Gemmatimonas phototrophica*, BChl orientation was fixed to absorb at 868 nm in their respective α or β subunits in a similar manner. They had specific His residues to stabilize the central porphyrin Mg^2+^ of BChl and Trp residues, which formed hydrogen bonds with the BChl C3^1^ keto group [29]. Alongside RC and LH1 protein interactions, additional polypeptides spanning the periplasmic side (RC-S) and the cytoplasmic side (RC-U) help solidify the complex, a feature absent in other RC-LH1 structures [29]. Thorough investigations are only beginning to be done for other AAPs [31]. More efforts towards this avenue are needed considering the fact that many AAPs also have LH2 with unusual absorbance peaks, indicating atypical pigment–protein interactions.

### 3.3. Light-Harvesting Complex 2 (LH2)

LH2 complexes serve as additional circular accessory antennae that surround the LH1-RC complex. Formed by subunits similar to those in LH1, they tend to be smaller in final size, usually grouping into octamers or nonamers [86]. The BChl within absorbs at different wavelengths than LH1, thereby allowing an even greater range of light usage, enhancing overall photosynthesis output. Typically, LH2 in purple bacteria will have two major peaks in the whole cell absorbance spectrum: at 800 nm and 850 nm [86]. They are due to the two BChl molecules associated with respective α and β subunit proteins. While most purple bacteria will have an LH2 to maximize light absorbance, in AAPs, it varies [2]. Many only have an LH1 or if they do have an LH2, it tends to be produced at lower numbers than in PNSB, with the 850 nm peak often appearing as a shoulder rather than a distinct peak in the spectrum (Figure 5, bottom right). The actual ratio of LH2 to LH1 has not yet been quantified for most AAPs, but in *Cg. litoralis*, it has been estimated as 1 LH2 per 3–4 LH1; however, this could be different depending on growth conditions [64]. Some AAPs have typical 800–850 nm LH2 peaks, albeit slightly shifted in certain cases, like in *Cg. litoralis* and *Polymorphobacter* strain FW250 (Table 2, Figure 5). However, the majority of AAP LH2 deviates from this norm.

### 3.4. Unusual Peripheral Light-Harvesting Complexes: LH3 and LH4

#### 3.4.1. LH3

This form has the typical bimodal shape seen in LH2 but is relatively blue-shifted compared to 800–850 nm and are in a variety of purple bacteria such as *Rhodoblastus acidophila* and *Rhodoplanes tepidamans* [87], which have 800–820 nm peaks, alongside *Allochromatium vinosum* and *Marichromatium purpuratum* with 800–830 nm instead [88]. Multiple different AAPs show LH3-like spectral features. *Sandarakinorhabdus*, *Porphyrobacter* and *Erythromicrobium* demonstrate absorptions at 800–837, 800–835, and 800–832 nm, respectively (Table 2, Figure 5), marking similarities to *A. vinosum* and *Mc. purpuratum*. *Pseudohaliea rubra*, a γ-*Proteobacteria*, has an LH3 bimodal at 804 nm and 821 nm (Table 2). Interestingly, it was produced in surplus relative to LH1, which is uncommon in AAPs [72]. Furthermore, it was expressed consistently unlike the LH3 in *Rhb. acidophila* [72,87]. Alternatively, β-*Proteobacteria Limnohabitans planktonicus* has an atypical LH with 799–813 nm peaks, somewhat similar to the 800–820 nm LH3 and to the peripheral antenna peaks in *G. phototrophica* (Table 2). In *Rhb. acidophila*, the 800–820 nm complex appears like a regular LH2, forming circular nonamers, that individually surround the LH1-RC [87]. In contrast, the *G. phototrophica* structure is unique by forming a secondary ring made of 24 αβ subunits that encircle the LH1-RC [29]. It would be insightful to determine if the *Limnohabitans* sp. LH2 resembles the typical octamer/nonamer arrangement or forms a concentric ring, like in *G. phototrophica*. Speculatively, the PGC in *Gemmatimonas* was obtained via lateral gene transfer from a β-*Proteobacteria* [89,90]; therefore, it is possible that phototrophic *Limnohabitans* has a similar peripheral complex organization.

Another variation in LH lies in the stable, consistently expressed LH4 found in select AAPs.

#### 3.4.2. LH4

There are multiple anoxygenic phototrophs with a peripheral antenna that displays an unusual single peak at ~800 nm. Called an LH4, this monomodal variant was first identified in purple sulfur *A. vinosum*, which was expressed only under low light conditions [91]. Later, a similar structure was detected in limited illumination for PNSB *Rh. palustris*, which was then characterized [92]. As of now, it has been identified in the following AAP: *R. mahoneyensis*, *Rb. denitrificans*, *Rb. litoralis*, *Rubrimonas cliftonensis*, and *Dinoroseobacter shibae* (Table 2, Figure 5) [6,59,80]. Unlike the LH4 in *A. vinosum* and *Rh. palustris*, AAPs appear to constitutively express LH4 as the exclusive peripheral LH. Although they share spectral features and the amino acid motif (F44L45) [59], there appears to be inherent differences in the stability of the structure. In *Rb. denitrificans,* it resisted detergent treatments during purification, while in *R. mahoneyensis*, it destabilized quickly [48]. Experimental analysis confirmed the LH4 in *Rb. denitrificans* has a less efficient carotenoid to BChl energy transfer, when compared to purple bacteria containing the same pigments [59]. *R. mahoneyensis* often produces spontaneous mutants, some of which make the LH4 without the RC and LH1 and were recently biophysically characterized [93]. The nature of AAP LH variations invites more study to comprehend their full effect and significance.

Although the LH3 and LH4 are distinct in spectral shifts and compositional features, they share the same role with traditional 800–850 nm LH2: to absorb and shuttle light to the RC. Therefore, all are functionally and structurally similar enough to be called LH2, as previously suggested [86]. To differentiate them, for nomenclature purposes, the two peaks can be used, providing a greater clarity than the current designations. No biophysical or structural studies have provided evidence that would require the classification of LH3 or LH4 as a ‘new‘ type of LH. Similarly, in LH1, despite even greater absorption variation among anoxygenic phototrophs, they fundamentally serve an identical role. LH1 and LH2 are distinguished by their association with the RC and arrangement, either directly encircling it as a 16/17-unit polymer (LH1) or serving as a more distant shuttler of *hυ*, which capture a different wavelength range and forms octamer/nonamer structures (LH2). One arguable exception would be the peripheral LH ring reported in *G. phototrophica*. Unlike a typical purple bacterium, its secondary ring, denoted as LHh [29], contains 24 subunits and surrounds the entire LH1-RC, forming a double-ring complex. Such a distinctive formation clearly deserves its own classification because it is a completely different arrangement to the LH2. This is supported by the other described factors responsible for the change in organization (Section 3.4.1), BChl absorbance peak shift (Table 2), and assembly coordination. Furthermore, due to its direct association, the energy flow from LHh to LH1 is faster than regular LH2 complexes measured in purple bacteria [29]. In contrast, the LHh also makes it difficult for the ubiquinone at Q_B_ to leave and return to the RC as the steric hindrance limits the ‘breathing motion’, which forms spaces for quinone movement in closed LH1 rings [29,31]. Cryo-EM investigations on AAPs with an LH4 and *Limnohabitans* with an LH3 to compare their respective purple bacteria and *Gemmatimonas* counterparts would help distinguish the key components that are responsible for the structural and spectral variation. Additionally, valuable insight on the different ways BChl and carotenoid pigments associate with the protein subunits and how this impacts their ability to absorb and transfer light energy would be acquired.

### 3.5. Photosynthetic and Accessory Pigments

#### 3.5.1. Bacteriochlorophyll

BChl *a* is the primary photosynthetic pigment in AAPs. Other anoxygenic phototrophs can utilize different BChls, with some producing multiple types simultaneously [94]. BChl *a* is incorporated into the RC, acting as the main catalyst of converting light into energy in the form of a transported e^−^, as well as in the LH complexes, shuttling *hυ* absorbed at different wavelengths towards the RC.

In almost all known BChl and chlorophyll (Chl) molecules, Mg^2+^ anchors the center of the tetrapyrrole ring. It was hypothesized previously that Mg^2+^ can be substituted with Zn^2+^, and this has been done in vitro [95]. However, it was not known to exist in nature until the discovery of *Acidiphilium rubrum* [96]. Some acidophilic AAPs indeed incorporate a Zn-containing BChl into their RC, with some producing it consistently (*Acidiphilium*) or conditionally, when zinc concentrations are increased in the habitat (*Acidisphaera*) [96,97]. Prior to its discovery in nature, artificially created Zn-BChl was shown to be more stable under acidic conditions than Mg-BChl *a* but just as efficient [96]. A noteworthy fact considering Zn-BChl has only been observed in acidophiles. There is an uncertainty about exactly how zinc is incorporated. Speculations are that Zn^2+^ either replaces Mg^2+^ or is inputted directly into the tetrapyrrole ring via a distinct chelatase [96]. The main absorbance peak of Zn-BChl (762.5 nm) has a blue-shift from regular BChl *a* (770 nm) in acetone:methanol (7:2) pigment extracts, and isolated reaction center peaks are also slightly shifted from typical AAPs and PNSB [96,97]. The most recently discovered anoxygenic phototroph with a Zn-BChl *a* is microaerophilic *Chloroacidobacterium thermophilum*. For this species, it serves an imperative role, acting as the P pair in the cyclic e^−^ transport pathway [98]. Study of acidic environments as a habitat for anoxygenic phototrophs is limited as they are usually in the dark or are very toxic, making it unsuitable for most phototrophs [99]. However, for AAPs that survive and thrive in higher oxygen levels, low pH habitats with access to light are potential sites for discovering new taxa. Such places include acidic bogs, marshes, lakes, and hot springs [99]. As many of them remain largely unexplored, it is very likely that there are other AAPs in nature that can utilize Zn-BChl. Through the investigation of new environments, there is the possibility of not only identifying new BChl but also new secondary pigmented molecules that participate in light absorbance.

#### 3.5.2. Carotenoids

Vibrant and boldly colored, carotenoids are very interesting natural elements known to have many, at times unexpected, functions [100,101,102,103]. As secondary pigments in photosynthesis, they assist in photon capture mainly in the 450–550 nm range and protect cells from photooxidative stress [2]. In contrast to other anoxygenic phototrophs, AAPs produce carotenoids in noteworthy excess, and they are always primarily responsible for the color of cells (Figure 5). They are either directly associated with the photosynthetic apparatus or randomly distributed across the membrane [2]. Estimates suggest a ratio of 1:8 to 1:10 of BChl molecules to carotenoids [2] in AAPs, although some have greater proportions of the primary pigment [25,27,104]. This contrasts with the purple bacteria, where the ratio is approximately 1:1 [2]. The difference has been attributed to the fact that AAPs grow aerobically while absorbing light energy, the combined causes of which amplified oxidative photodegradation to cells versus anaerobic conditions [2]. Therefore, carotenoids are produced in surplus not for photosynthesis needs but to mitigate the negative effects.

In the photosynthetic apparatus, carotenoids aid in light capture, but the degree of participation varies [48,49,64,105,106,107] and depends on the types produced [106,107]. For example, spheroidenone synthesized in *Rhodobacterales* is directly involved in light harvesting [108] as well as bacteriorubixanthinal, zeaxanthin, and β-carotene for *Sphingomonadales* [32,107,108]. The C30 carotenedioate and its diglucosylester form participate in light transfer in *Rc. thiosulfatophilus* [105,109]. Alternatively, they also perform a structural and assembly role. Previous studies with carotenoid-free mutants of *Cereibacter* (formerly *Rhodobacter*) *sphaeroides* and removal of carotenoids from *Rhodobacter capsulatus* membranes, showed that cells were unable to assemble LH2 properly and the protein subunits were rapidly degraded [110,111,112]. Analysis of the *G. phototrophica* photosynthetic complex via cryo-EM showed that carotenoids were integral to the structure of the LH complex(es) [29] and may aid in assembly [113]. Another major role is sequestering singlet oxygen or BChl triplets produced during pigment synthesis [2,106].

Carotenoids in the membranes that are not associated with photosynthesis [105,109] are involved in singlet oxygen scavenging of radicals and protect cells from light damage during prolonged, intense illumination [48]. Such is the case for erythroxanthin sulfate, the major pigment of *Erythrobacter* and *Erythromicrobium* [107].

Although some AAPs only have one dominant type, while others make more than ten [2,109], the accessory pigment composition is shared or very similar among species and genera [14]. Nostoxanthin is common in all *Citromicrobium* [114]. Spirilloxanthin is the primary carotenoid in the *Halieaceae* (NOR5/OM60 clade, γ-*Proteobacteria*) as well as *Sphaerotilaceae* (*Rubrivivax pictus* and *Rt. depolymerans*, β-*Proteobacteria*) [27,115,116]. Additional examples have been provided above.

While pigments are necessary for light-harvesting and initiation of photosynthesis, e^−^ carriers are essential to the completion of the cycle and ATP synthesis.

### 3.6. Electron Transporters: Quinones and Cytochromes

With ubiquinones and cyt (in particular cyt *c*_2_), participating in both photosynthetic and respiratory e^−^ transport chains, there is no doubt that they influence the efficiency of both processes. Furthermore, the e^−^ carriers may offer clues on how AAP manage to use each system simultaneously. Obviously, a delicate balance must be held as there is a limited availability of e^−^ donors and acceptors for these activities. An increase in oxidation of organic carbon could limit the availability of e^−^ for photosynthesis and an excess activity of the light-dependent reactions may hinder the use of e^−^ for other cellular activities or induce overproduction of toxic oxygen radicals [44]. It is possible that some may have different quinones and cyt performing respiration and photosynthesis, respectively. However, that has not been proven, and some only produce a single cyt *c* or ubiquinone with the appropriate redox potential that can fill the role in e^−^ transportation [44,47].

Quinones are possibly one of the key reasons why AAPs require aerobic conditions for photosynthesis. They are lipid-soluble e^−^ carriers that move across the membrane to transport e^−^ from the RC to the cyt *bc*_1_ complex. Ubiquinones at the Q_A_ site in AAP usually have a redox midpoint potential 65–120 mV higher than PNSB (Table 1). As a result, at this site, they would be fast if not immediately and completely over-reduced under anaerobic conditions, making it impossible for them to accept e^−^, hence halting the pathway altogether [2]. PNSB have an alternative quinol oxidase pathway to keep it in the correct redox state when oxygen is present, but not AAPs [2]. Typically, the midpoint potential of binding site Q_A_ in AAPs is between +5 and +150 mV, but in *Rb. denitrificans*, the midpoint potential is −50 mV and in *P. meromictius*, it is −25 mV, which despite being negative, is still not sufficient [43,48,54]. Their respiration pathway is not functional when oxygen is absent, therefore the quinone pool would become over reduced, as there is no mechanism to regulate the redox state regardless of the Q_A_ value [54]. Alternatively, even with a typical Q_A_ midpoint value, *R. mahoneyensis* uses its photosynthesis only semi-aerobically because the quinone (+94 mV) and its RC-bound cyt *c* (+277 mV) form a narrow redox potential range where e^−^ transfer through the entire cyclic pathway is possible [48]. As such, quinones do not provide a simple answer but likely are a component that corresponds to the ability to photosynthesize in exclusively aerobic conditions [22].

Soluble cyts are involved in the transfer of e^−^ from the cyt *bc*_1_ complex to the RC via the periplasmic space and can be associated with the RC to varying extents (Section 3.1). So far, cyt *c*_2_ has been the only one found to complete cyclic e^−^ transfer in AAPs [2]. In *Cg. litoralis*, a gene for cyt *c*_2_ was not annotated, suggesting that another cyt or a high-potential iron-sulfur protein serves that role in the pathway [25]. In some species, only a single soluble cyt *c*_2_ is identified, like in *Er. hydrolyticum* and *S. sibiricus*, therefore making this a shared e^−^ carrier for both respiration and photosynthesis [2]. Others have multiple cyt *c* with suitable redox potentials [47,105].

Most of the components described here in Section 3, which are exclusively used in photosynthesis, are organized in a superoperon. At this scale, major differences in the organization and composition are found, which are described in the next section.

## 4. The Photosynthesis Gene Cluster

### 4.1. Composition and Arrangement

**Figure 6:**
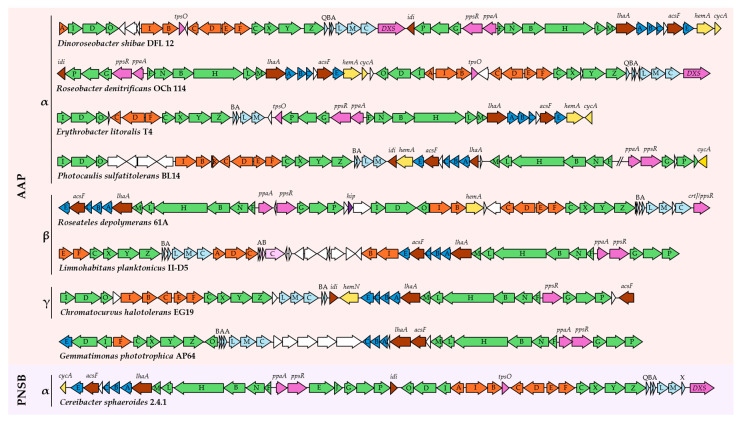
The photosynthetic gene cluster of selected AAPs and PNSB. Genes are colored as follows: green (*bch*), orange (*crt*), light blue (*puf*), blue (*puh*), yellow (*hem* and *cyc*), purple (regulatory genes), brown (other photosynthesis genes), pink (*puc*), dark purple (*hip*), and white (hypothetical proteins, uncertain or unrelated genes). Those in *Pseudomonadota* are labeled with their class. GenBank genome accession numbers of strains are the same as those in Supplemental Table S1.

In anoxygenic phototrophs, the sequences required exclusively for capturing and harnessing light energy are primarily found within a superoperon called the PGC (Figure 6) [117]. It encodes genes for the synthesis of BChl (*bch*, in four conserved regions: *bchFNBHLM*, *bchCXYZ*, *bchIDO* and *bchGP*), carotenoids (*crt*), RC and LH1 proteins (*puf* and *puh*), and some additional genes required for expression, regulation, and function (*lhaA*, *acsF*, *ppaA(aerR)*, *ppsR(crtJ)*) [114]. Most AAPs share these genes in common as they are essential to photosynthesis. The PGC size of most AAPs is comparable to that in PNSB, from 45 to 50 kbp [108,118], except in *Sphingomonadales*, in which it is a lot smaller, approximately 39 kbp [108]. The G + C content of the PGC is typically similar to the overall genome, suggesting the stability and homogeneity of this superoperon with the chromosome. It strongly supports the idea that the PGC is a component that evolved with AAPs in their distinct lineages, and if horizontal gene transfer (HGT) occurred, it was an ancient event [108].

There are certain PGC traits that distinguish AAPs from PNSB. Features in common for the majority of AAPs are the absence of *bchEJ* in the PGC (*Rhodobacterales*) or the genome all together [108,119]. The loss has been hypothesized to be a result of the genes having little to no function or benefit in aerobic environments [32,120]. BchE is the anaerobic version of enzyme Mg-divinyl protochlorophyllide, which uses water as a substrate, while AAP primarily uses the aerobic form called AcsF that uses oxygen instead [32,121]. For BchJ, the role it serves is still unclear [122]; therefore, the impact on AAP is unknown. Another difference to PNSB is the absence of *pufX*, which plays an important role in the formation of proper LH1-RC complexes in some PNSB [108,123]. Interestingly, in *Rhodobacteraceae*, *pufX* and *pufC* are located in the same region of the PGC, and no known anoxygenic phototroph has both. With *pufC*-containing members forming the oldest branches phylogenetically, it is implied that *pufX* may have been a replacement of *pufC* in certain phototrophic lineages [124]. An enzyme that catalyzes a step in BChl synthesis called protochlorophyllide has a variant that is more common in AAP and another that is associated with PNSB. The light-dependent form is believed to occur as a result of HGT from an oxygenic phototroph to *Pseudomonadota* [89]. This form is primarily found in AAPs, likely because it is more tolerant to oxygen unlike its dark-operative counterpart in PNSB [89]. Being suited for aerobic conditions, protochlorophyllide has its benefits, but since it is light-dependent, it could also limit the amount of BChl that can be produced at a given time.

There are still many other differences between AAP, such as the presence and absence of certain genes. In *Bradyrhizobium,* PGC contains a bacteriophytochrome and two different *ppsR* genes: one that activates the PGC and the other that represses it [118,125]. Another variation is the presence of *pufC*, which encodes the optional cyt *c* subunit of the RC [108]. In *Halieaceae* and PNSB, *crtJ*, which represses BChl, carotenoid, and LH2 gene expression under aerobic conditions, is present [108]. The gene is obviously more aerotolerant or performs a different role from that in typical PNSB since all *Halieaceae* are AAPs. Such change in function from the original description has been reported before. In *Rhodospirillum centenum*, *aerR* activates the PGC, unlike in typical PNSB, where it is usually a repressor [126]. Gene *pufQ* is absent in *Halieaceae* and *Sphingomoadales* [108], which regulates porphyrin flux to either haem or BChl production and thus is important for maintaining stoichiometric balance of components involved in both respiratory and photosynthetic energy production [127]. Many AAPs have *ppsR* and *ppaA*, monitoring photosynthesis regulation in response to light and oxygen levels [32].

Carotenoid genes are by far the most variable trait in the composition of the PGC (Figure 6). In *Brevundimonas subvibrioides*, *crtYIBWZ* is not in the PGC [119]. In *Rhodobacterales*, *crt* genes are all situated in the PGC while some are not in *Sphingomonadales* (*crtIBEYZ*) and *Halieaceae* (*crtD*) [108]. Both *Sphingomonadales* and *Halieaceae* also do not contain *crtA*, a requirement for spheroidenone synthesis [108]. *crtYZ* genes, which form the initial steps for pigment synthesis in *Erythrobacter* and *Citromicrobium*, are not associated with the PGC [108]. Therefore, regulation is likely independent from those in the PGC [108], supporting previous physiology and biochemistry experiments for *Erythrobacter* which show their lack of participation in photosynthesis [107,109].

Another difference among AAPs lies in the organization of the superoperon. There are certain genes that appear to cluster together regardless of the species and its phylogenetic origin: *bchFNBHLM-lhaA-puhABC* and *crtF-bchCXYZ*. However, the orientation and order of the components vary, creating four different subtypes well described by Zheng et al. [108]. In general, the representatives that are closely related have a conserved arrangement [108].

While most AAPs have PGC on the major chromosome within a single cluster, this is not necessarily so for all. In *Ph. sulfatitolerans* (Figure 6), *Ac. multivorum*, *Loktanella vestfoldensis* SKA53, and a few others, the superoperon is split [73,108,119,128]. For *Methylorubrum extorquens*, a methylotroph associated with AAPs, the PGC has four different segments that are separated anywhere from 291 kb to 2058 kb [119].

Alternatively, the PGC can be located on a plasmid. This has been observed in *Rb. litoralis*, with only two genes, *bchEJ*, located on the chromosome [119], as well as *Shimia ponticola* [129], *Algirhabdus cladophorae* [130], *Sulfitobacter noctilucicola, Nereida ignava*, *Tateyamaria* sp. ANG-S1, and *Oceanicola* sp. HL-35 [124], where all PGC genes are plasmid-bound. This implies the extrachromosomal mobile elements can serve as a vector for HGT, and the entire PGC unit is more flexible than initially assumed. Interestingly, in *Rb. litoralis*, PGC expression in response to light and nutrient deprivation matches what is in other AAPs (Section 5.1 and Section 5.2), suggesting it is still regulated to match the cell’s needs [131]. There are two possible reasons: (1) the PGC is primarily controlled by major cell regulators that can recognize target sequences or (2) it is excised from the main chromosome and is therefore still recognized by photosynthesis-specific regulators.

When present in AAPs, the LH2 genes (*pucABC*), like in most other proteobacterial anoxygenic phototrophs, are not situated in the PGC [108]. The separation of LH2 allows it to be independently regulated and expressed when beneficial [25,132]. A notable exception is *Limnohabitans* (Figure 6) [71]. Most *Spingomonadales* do not have LH2 genes [108].

Non-proteobacterial AAP *G. phototrophica* is organized like in other purple bacteria (Figure 6) [133], but for *Vulcanimicrobium alpinum*, the genes are randomly distributed throughout the genome [30]. Although it is the only known anoxygenic phototroph to have such a distribution of the photosynthesis genes, it is very likely that through sequencing, more distinct organizations, or lack thereof, will be found.

### 4.2. Hidden AAP

As whole genome sequencing over time has become more accessible and affordable, potential AAP species are being identified through the discovery of the PGC in the chromosome, including those initially described as chemoheterotrophs. While the presence of PGC alone cannot confirm the photosynthetic activity in vivo, it does provide a direct reason to investigate and find out [71]. Even when expression cannot be induced in vitro, there is still a chance it could be used, but only under appropriate conditions. An example of tightly regulated and constricted use of photosynthesis is *Aquincola tertiaricarbonis* strain L108. When provided minimal 2-methyl-2-propanol as the carbon source, pigment and photosynthetic complex production occurred [134]. Alternatively, there is a chance that despite the PGC’s presence, the organism is still unable to use it advantageously. Possibly, mutations in key genes render the product non-functional or other regulatory components that induce its expression are absent. However, the identification of the PGC can still provide valuable insight into evolution. By comparing the sequences of key genes, phylogenetic relations can be assumed and support speculation on photosynthesis emergence in certain groups or their loss (Section 4.3).

DNA sequencing has shown that the prevalence of photosynthesis might be a lot greater than previously estimated. Many species described as non-pigmented or not investigated for light-harvesting properties were confirmed to contain genes for photosynthesis. Some methylotrophs like *Methyloversatilis* spp. [115] and *Methylocella silvestris* [119] have a PGC, but there is no proof that it is expressed and used. Metagenomic sequencing and the assembled genomes (known as MAGs) have also helped uncover a broader extent of potential AAPs. For example, the PGC was found in *Myxococcota candidati* taxa MAGs, some of which were tentatively noted to be obligate aerobes and, therefore, potentially AAPs [37]. While better studied for their distinct predatory lifestyle, photosynthesis may be advantageous for *Myxococcota* in prey/nutrient-limited conditions [37].

Gene contents alone do not confirm the light-harvesting ability and conversion of photons into universal biological energy ATP. However, by exploring which genomes do or do not contain this, their relation to others can provide insight into the evolution of photosynthesis, the variations in its usage, expression, and distribution, especially with organisms of particularly distinct lifestyles or capabilities, like *Myxoccoccota*.

### 4.3. Insights into Evolution

Through the different approaches, speculations, perspectives, and genes analyzed, a clearer but still complicated picture of AAP evolution is emerging. In the pioneering work of Carl Woese based on 16S rRNA sequencing as a representative conserved gene to explore the phylogenetic distribution and evolution of life, he proposed that the ancestor of *Pseudomonadota* was a phototroph that deviated over time with photosynthesis being lost at different lineages [135]. In recent years, his hypothesis on photosynthesis in *Pseudomonadota* has been questioned, as results have shown that it is unable to fully account for the phylogenetic and photosynthetic diversity of AAPs. On the one hand, the PGC is very conserved in certain parts indicating an ancestral lineage, while on the other, similarities to distantly related species or fragmentation of the cluster suggests that HGT likely played a role. Thus, the most appropriate explanation for AAP evolution is likely not as simple as previously interpreted. It probably comprises members with inherited photosynthesis and those that laterally acquired the trait. There are many ways genetic information exchange could have taken place, such as conjugation via the type IV secretion system, plasmids, gene transfer agents (phage-like particles), transduction, and transformation [124,136]. Here, we discuss how genomic analysis has enriched our understanding of different AAP groups, especially regarding the suspected mechanisms of HGT and events of photosynthesis evolution that have contributed to its phylogenetic dissemination.

Studies investigating specific photosynthesis genes have indicated that AAPs as a whole are a product of multiple lineages. In a large project that looked at the relation of *pufLM* sequences in *Pseudomonadota*, the orders *Chromatiales, Rhodobacterales,* and *Sphingomonadales* were defined as major groups distinct from other α-*Proteobacteria*, β-*Proteobacteria*, and *Caulobacterales* (*Br. subvibrioides*). Therefore, they concluded that there must have been several independent transitions from an anaerobic to an aerobic photoheterotrophic lifestyle that took place in this phylum [137]. Whether this may have happened before or after differentiation of lineages occurred is still uncertain.

Another study explored the sequences of an enzyme imperative to all types of BChl synthesis, chlorophyllide reductase (*bchXYZ*). Here, it was suggested that the different AAP groups were a result of multiple events rather than one, with *Rhodobacterales* being the youngest [90]. The idea somewhat aligns with previous work that suspected this order forms a distinct group from other typical α- and β-*Proteobacteria* AAPs, potentially because the transfer of the PGC was via plasmids [137]. Maybe the plasmid-driven dissemination of the PGC is relatively recent in the grand scheme of anoxygenic photosynthesis evolution and phylogenetic distribution.

The same study also postulates that *bchXYZ* in *Gemmatimonas* is closely aligned with β-*Proteobacteria* [90]. This connection was also made in another report, where its light-dependent protochlorophyllide oxidoreductase is also most similar to β-*Proteobacteria* [89]. Considering the PGC of *Gemmatiomonas* is unregulated [133], the idea that it was acquired through HGT from a β-*Proteobacteria* is likely. Furthermore, the attainment of photosynthesis by β-*Proteobacteria* was hypothesized to occur via an α-*Proteobacteria* donor [89]. Therefore, the *Gemmatimonas* PGC may be a result of multiple HGTs.

The ancestor of BChl-containing *Bradyrhizobium* is suspected to be a heterotroph, and AAP presence in different lineages is a result of multiple independent events of HGT [118]. Contrastingly, for *Acetobacteraceae*, comparison of photosynthetic genes and respiratory enzymes within the family was used to conclude that the ancestor must have been a phototroph, supported by the identification of remnant carotenoids in non-phototrophic members alongside PGC-containing groups, forming basal clades within the family [51]. Additionally, no photosynthesis-accommodating plasmids were found in the genomes, suggesting that AAPs in this group are likely a product of evolutionary adaptation to oxygenated environments [51].

In *Rhodobacteraceae*, photosynthesis was probably acquired through HGT based on the sporadic appearance throughout the family, incongruence between core genome and PGC-based phylogenetic trees as well as the presence of the PGC on plasmids of *Sb. guttiformis*, *Rb. litoralis*, *Sb. noctilucicola*, *N. ignava*, *Tateyamaria* sp. ANG-S1, and *Oceanicola* sp. HL-35 [124]. Whole genome sequencing showed that the earliest branches are composed of heterotrophs, further supporting this hypothesis [124]. While plasmids appear to be one possible mechanism of HGT in this family, another may be conjugation through Type-IV secretion systems [124]. The minimal differences in the PGC hint that this could have been acquired relatively recently, corroborating with a suggestion that *Rhodobacterales* may be the newest AAP order [90]. In contrast, others have thought the *Sphingomonadales* are the latest group that emerged, due to the lack of LH2 in most species and simplicity of their PGCs [114].

Evidence of HGTs in *Sphingomonadales* has been noted. In *Erythrobacter* strain AP23, the PGC likely originated from *Citromicrobium* due to its alignment and also because the strain LAMA 915 (with a 99.5% 16S rRNA similarity) does not have genes for photosynthesis [138]. In *Erythrobacter* strain HWDM-33, the PGC was located near a Type IV secretion system, showing the potential to be mobilized into other genomes [138]. An uncommon case is *Citromicrobium* sp. JL354 and JLT1363, which shares a 98.1% 16S rRNA similarity [114]. Uniquely, JL354 had 2 PGCs; one is complete and the other only contains *pufLMC*-*puhABC*. Strain JLT1363 had no photosynthesis genes. When comparing the surrounding region of the complete PGC in JL354 to JL1363, they were the exact same except JL1363 had only a single gene, not an entire superoperon [114]. The PGC-based phylogenetic trees aligned well with those based on 16S rRNA for JL354, therefore indicating that photosynthesis was acquired a long time ago and that JLT1363 lost their genes. Interestingly, JL354 also represents a potential case of HGT. The GC content of the incomplete PGC (66%) was higher than that in the genome (62%), and the sequence similarity between replicate genes was less than 80%, eliminating the possibility of gene duplication [114]. Furthermore, its location in a putative integrative conjugative element provided insight on the presumed mechanism of lateral gene transfer and the *pufC* being most similar to *Fulvimarina pelagi* suggests that it is the original host of acquired genes [114]. This scenario serves as a case study of photosynthesis incongruity in AAP lineages, showing that the PGC can be gained and just as easily lost.

Overall, the PGC has features that are well-conserved, such as *acsF* [121], supporting the idea of vertical evolution alongside more variable characteristics, including organization, composition, and genome location, which indicate that lateral gene transfer occurred. Evidence suggests that the evolution of AAP photosynthesis in *Pseudomonadota* involves both inherited and obtained traits to varying degrees, depending on the lineage as discussed. Thus, the AAP PGC is a microcosm of the complicated nature of evolution, where the acquisition and loss of genes is as variable as the potential physiological capabilities.

## 5. Environmental Influences on Photosynthesis Activity

A variety of physical parameters play a role in the usage of photosynthesis by AAPs. One study compared the photosynthetic complex production and size among different *Pseudomonadota* AAPs [58]. AAPs with no accessory antennae had a smaller photosynthetic unit size than those with LH2. *Rb. litoralis* had an overall photosynthetic complex production comparable to PNSB [58]. The other AAPs had ten-fold less, matching known BChl production calculated for PNSB *Cb. sphaeroides* (20 nmol of BChl/mg of cells) and AAPs (0.7–2.0 nmol of BChl/mg of cells) (based on dry weight) [2]. This exemplifies the variance in regulation within AAPs even when grown in the same conditions. Differences are amplified when certain environmental variables are also accounted for and sometimes required for even minimal expression [18,25,26,71]. While the anoxygenic photosynthesis requires significant amounts of energy to maintain, the yields in AAPs are small, providing 10–50% of the cellular energy [44]. Nonetheless, AAPs are common phenomena, showing that it is indeed beneficial. Here, three of the most well-studied parameters (light, oxygen, and nutrients) are highlighted to show how they affect photosynthesis expression.

### 5.1. Light

Light inhibits BChl production in AAPs [24,26,27,96] especially with greater intensities [25,104]. The pioneering study that discovered this controversial, unexpected influence was a chemostat experiment on *Er. hydrolyticum* conducted by Yurkov and van Gemerden, where the levels of BChl were monitored during growth in (1) complete darkness, (2) complete light, and (3) a light/dark regimen [24]. Here, BChl was made exclusively in the dark [24]. Furthermore, in dark/light treatment, it was synthesized at higher rates and ultimately led to greater growth versus the alternate conditions, showing the benefits of photosynthetic productivity [24]. This finding was fundamental to understanding AAPs. It proved undoubtedly that the group used light energy and benefitted from the process, despite hurdles in BChl production. Additionally, the limitation is backed by logic: BChl synthesis creates oxygen radicals and in conjunction with light and atmospheric oxygen, damage to cells would be intensified [2,44]. Despite this, the overall amounts of BChl are still sufficient to keep up with cell division, which would dilute the amount of the photosynthetic apparatus over time. Energy gained from light allows them to redirect organics to anabolic synthesis instead of ATP production [44,139,140]. As a result, during light periods, AAPs have a decreased respiration rate and lower oxygen uptake [11,104,139,141]. Further exploration of the regulation in response to light/dark growth cycles was done by performing a transcriptomics experiment on *D. shibae* [142]. Over time in light, O_2_ respiration genes were upregulated as the photosynthesis energy yield decreased. Interestingly, synthesis of pigments did not start until 4 h into the dark cycle, while exposure to light immediately halted expression [142]. Similar observations have also been detected in phototrophic rhizobia [33]. The delayed response of photosynthesis gene expression in the dark is a survival strategy; by directing energy towards replicating in the early dark period, a greater number of cells can be formed, which will later synthesize the photosynthetic apparatus, optimizing both survival and cell productivity [142].

In *D. shibae* [26], *Cg. litoralis* [25], and *C. halotolerans* [116], continuous production of photosynthetic pigments in the presence of light was observed. In *Cg. litoralis*, it was estimated that growth in light yielded 2.4 times more ATP than exclusively heterotrophic growth in the dark [25]. For *D. shibae*, consistent expression in light may be a result of culture shadowing, providing sufficient darkness for BChl synthesis to continue [26]. In their natural environment, the effect could be limited in the form of a biofilm [26]. It also serves as a suitable explanation for the contradiction to the foundational work mentioned above, where pigment synthesis was halted in light [142]. Alternatively, some aerobic phototrophs require initial exposure to light before pigment synthesis begins in next dark phase, such as in *Bradyrhizobium*, *Methylobacterium rhodesianum*, and *M. extorquens* AM1 [35,118,143]. Therefore, not all AAPs are regulated as strictly to light and have other techniques to combat photooxidative damage.

Natural habitats will have variable durations of light periods, and AAPs must adapt to this. An extreme example is *Sediminicoccus* sp. strain KRV36, which continuously produces the photosynthetic apparatus in the light and contains chromatophores, a feature rarely seen in AAPs [43,46]. It was isolated near the Arctic Circle, where there is a continuous light period in the summer. The strain is well-adapted, constantly synthesizing BChl and estimated to contain ~40,000 RC-LH1 complexes per cell, surpassing amounts found in some PNSB [46]. Furthermore, when grown in light, photosynthesis comprised 60% of the energy acquired [46]. Photooxidative stress in KRV36 is combated with a high carotenoid content, enhanced expression of oxidative stress response genes, cell aggregation, and biofilm formation [46].

Light intensity affects the expressed features of the photosynthetic apparatus. In *Rb. denitrificans* and *Rb. litoralis*, LH2 production is greater in low light, maximizing the photosynthesis capacity. At higher illumination, the number of photosynthetic units increases, but they are smaller in size [58,144]. This strategy was suggested to help combat photooxidative damage by limiting the number of photons directed towards the RC, while maximizing energy yield [58]. Similar adaptations have also been observed in *D. shibae* [140]. Light exposure also increases the temperature on the cell surface membranes, as part of the absorbed energy is dissipated as heat. It appears that for this reason the photosynthetic complex in tested AAPs functions most optimally at slightly elevated temperatures from their growth conditions [145].

Certain light wavelengths impact the photosystem expression differently. *D. shibae*, *Rb. denitrificans*, *Halieaceae* AAP, and *Mb. radiotolerans* have photoreceptors that respond to blue light and indirectly affect PGC expression [108,146,147,148]. In *D. shibae*, the protein called LdaP is an antirepressor that is only active in the dark where it binds and halts PpsR activity, thereby allowing photosynthesis genes to be transcribed [146]. Why do AAPs have a sensor for blue light in particular? It is likely because this form is able to penetrate the deepest in aquatic habitats, such as the ocean, allowing them to sense if usable light is present [149]. Although energy acquired by light for AAPs is described as supplemental, in certain habitats that are limited in nutrients like open marine waters, it is likely essential for survival. Therefore, these sensors improve photoadaptation, allowing cell resources to be used efficiently. In the limited work done on photosensors in AAPs, those which respond to blue wavelengths are the most studied. However, they are not the only ones. For example, *Mb. radiotolerans* also has a red light sensing biliverdin-binding bacterial phytochrome [148]. Further work on the photosensing abilities of AAPs is needed to understand the full effects of light on this group. Overall, the response to light in AAPs varies, with some having very tight control as classically described while others may have more lenient regulation to accommodate their environment, as is the case for *Sediminicoccus* sp. strain KRV36. The next critical factor discussed for photosynthetic apparatus expression is also imperative to their survival: oxygen.

### 5.2. Oxygen

Despite the seemingly contradictory naming, oxygen is essential to anoxygenic photosynthesis in AAPs [47,54,150]. As described (Section 2 and Section 3.6), atmospheric levels create the necessary environmental redox conditions for the apparatus to function properly. Furthermore, most AAPs are unable to grow or survive if it is absent, as they depend on aerobic respiration for ATP synthesis. Some may be tolerant to lower concentrations such as *Rb. litoralis* or *Erythrobacter* sp. NAP1, but photosynthetic apparatus production and therefore the potential energy yield decreases [58]. However, there are some exceptions. For *R. mahoneyensis*, microaerobic environments are optimal for photosynthesis [48]. It is not alone, as *Rb. litoralis* [58] and *Cg. litoralis* [25] also have increased photosynthetic productivity semiaerobically. Although the ability to use photosynthesis at lower oxygen levels is uncommon in AAP, it could be a useful adaptation. For example, the rapid consumption of oxygen by heterotrophs with faster metabolism, could lower its availability for AAPs and if their apparatuses can accommodate this redox change, then they can survive using photosynthesis as an alternative route [48]. In *D. shibae*, it has been observed that photosynthesis-related genes are amplified, some up to 17-fold, when grown in dark, oxygen-depleted conditions, suggesting that it is used to make up for limited aerobic respiration [151]. Additionally, semi-aerobic conditions can help AAPs circumvent the problem of radical formation and singlet oxygen damage during BChl synthesis, making the process a more useful option, as less effort is needed to mediate the negative side effects. With these benefits, it would be logical to assume that the tolerance of the photosynthetic apparatus and AAPs themselves to lower oxygen levels is a more common phenomenon. However, the inability of most to grow at below atmospheric levels and the heavy reliance on respiration makes this a low probability. While this is a stressor that many AAPs are unable to overcome, photosynthesis has been proven to help them survive in carbon-limited environments.

### 5.3. Nutrients

It has been thought that a key advantage of AAP’s ability to use photosynthesis is that it can allow them to be less reliant on available organics, especially in periods of starvation. In oligotrophic environments, it is hypothesized that they partially substitute aerobic respiration with photosynthesis [44]. This capability is helpful in places, such as the open ocean, where nutrients are limited [26,44]. Various studies have indeed found that AAPs employ photosynthesis in oligotrophic conditions or during survival modes [26,104,141,152]. In fact, such a response has been observed even without light stimulus, where starvation alone triggers increased photosynthetic pigment production in *D. shibae* [26]. In contrast, others like *Hoeflea phototrophica* and *Stappia* sp. DFL-11 required light stimulation alongside starvation to induce BChl synthesis once nutrients and dark conditions were restored [26]. In *Rt. depolymerans*, it was found that the depletion of carbon for respiration is critical for photosynthesis activation [153]. Specifically, an RpoH sigma factor called SP70, which is expressed by *Rt. depolymerans* cells during starvation, is essential for photosystem production [153].

Nutrient deprivation is not the only trigger of photosynthesis. The type of carbon source influences expression as well [14,33,116,139]. Strict regulation, depending on organic availability and type, ensures light energy is used only when needed, therefore optimizing productivity. When nutrients are abundant, AAPs can use aerobic respiration for maximal yield. When carbon availability is low, photosynthesis-generated ATP can help with essential cell activities, thereby allowing cells to preserve needed resources [139]. For AAPs, such conditions are likely more common, therefore the ability to employ photosynthesis is not only opportunistic but also critical to survival. Exploring the factors that regulate this dynamic are one of many exciting future research prospects.

## 6. Future Directions, Perspectives and Conclusions

AAP knowledge is advancing as new techniques are being implemented for their study. The use of transcriptomics has allowed AAP photosynthesis regulation to be uncovered, providing incredible insights and potential new avenues of research. Metagenomics provides a fascinating opportunity to identify novel taxa [37] and potential growing conditions needed for culturing a variety of species. For photosynthetic apparatus structural analysis, cryo-EM has started to be used and has already given incredible results [29,31,46,64]. There is no doubt that by continually developing the methods of study, it will bring us closer to fully understanding the AAP puzzle.

One aspect that requires more attention is their regulation. While we understand the major factors that influence photosynthesis expression (Section 5), exactly what is involved with coordinating this response is still limited. The roles of RNA chaperones, sRNAs, asRNAs, sigma factors, photoreceptors, and other nontraditional regulators have been found for PNSB [154,155,156,157,158,159,160,161,162] and, therefore, are likely involved in AAP as well. Current work is limited, but a blue light-responsive antirepressor of *ppsR* called LdaP has been described in *D. shibae* (Section 5.1) [146]. Greater focus on this and the role of sensors could help identify activators, repressors, and additional factors exclusively involved in AAPs, further differentiating them from other anoxygenic phototrophs. Additionally, if some known regulators of anaerobic anoxygenic photosynthesis work differently in AAPs, this brings a clue into how the apparatus was adapted to increasingly oxygenated environments during the course of Earth’s history.

Regulation is one of many facets of AAP research that should be explored. The emergence of dual photosynthesis as a mode of metabolism is also noteworthy. The discovery, identification, and validation of *Sphingomonas* sp. strain AAP5, which conducts rhodopsin-based photosynthesis in addition to the BChl type was recently described [163,164]. Temperature and light intensity were the major factors influencing expression of both versions of light energy capture, reflective of the isolates’ natural habitat, a mountain lake [164,165] Based on MAGs and sequencing of gDNA, dual photosynthesis may be more common and phylogenetically widespread [166]. Further investigation of the two-system coordination aside from strain AAP5 can provide insight into how they work in other habitats. Due to the simplicity of the rhodopsin, both physically and in its genetic composition, there is not sufficient support to classify dual phototrophs as distinct from AAPs, but rather as a special subgroup.

As alluded previously, those that fit the minimum criteria of being an AAP, that is conducting anoxygenic photosynthesis in aerobic environments, form an incredibly large group. This includes those not traditionally associated with AAPs, like methylotrophs and rhizobia, and species outside the *Pseudomonadota* phyla, like *G. phototrophica*, *V. alpinum*, and *Ch. thermophilum* [9,10,33,150]. *G. phototrophica* has a PGC very similar to typical AAPs, but can only grow and therefore use photosynthesis in microaerophilic conditions and also have a unique LH structure (Section 3.4.1) [29]. Similarly, *V. alpinum* also requires below atmospheric oxygen levels for survival, but does not have a PGC, with all its photosynthesis genes scattered throughout the genome [30]. In *Acidobacteriota*, the unique *Ch. thermophilum*, does not fit clearly in any category of anoxygenic phototrophs [36]. It uses four different primary photopigments (Zn-BChl *a*, BChl *a*, BChl *c*, and Chl *a*) and produces chlorosomes, with the only clear AAP feature being the use of anoxygenic photosynthesis microaerobically [98,167]. As such, the question of whether or not they should be classified as AAP emerges. Although they are from different phyla, *G. phototrophica* and *V. alpinum* share the features in absorbance spectra and gene content with most AAPs, and their microaerophilic requirement is similar to *R. mahoneyensis* (Section 5.2). Phototrophic *Gemmatimonas* species may have even gotten their apparatus from *Pseudomonadota* (Section 4.3), so the similarities are enough to group them with AAP. On the other hand, *V. alpinum* photosynthesis genes do not show a significant relatedness to any cultured anoxygenic phototrophs. Therefore, it could represent a new group, but there is not enough evidence to show that its apparatus functions differently from AAPs. The only certain organism that should be excluded from the group is *Ch. thermophilum*. Biophysical testing has shown that it fundamentally operates distinctly from all AAPs described [98,167]. As such, AAPs can be classified as a multi-phyla group, making it the most phylogenetically widespread group of bacterial phototrophs.

Understanding the photosynthetic apparatus in its entirety, from the variable phenotypes to genetic components, is essential for uncovering the complex nature of AAPs and defining what truly sets them apart. As we have shown here, the known members are remarkably diverse in their photosynthetic protein–pigment complexes, and the resulting work has revealed capabilities that were previously not associated with anoxygenic phototrophs. Continued work with this fascinating group will without a doubt unveil new unexpected possibilities.

## Figures and Tables

**Table 1:** Midpoint potential of binding site Q_A_ in various AAPs and PNSB.

Species	Q_A_ (mV)	Reference
*Erythrobacter litoralis*	+150	[2]
*Erythromicrobium ramosum*	+80	[2]
*Sandaracinobacter sibiricus*	+5	[2]
*Blastomonas ursincola*	+25	[2]
*Porphyrobacter meromicticus*	−25	[48]
*Roseobacter denitirificans*	−44	[23]
*Roseobacter denitirificans*	−50	[43]
*Roseicyclus mahoneyensis*	+94	[48]
*Chromatocurvus halotolerans*	+85	[50]
*Rhizobium* strain BTAi1	−44	[55]
*Cereibacter sphaeroides*	−70	[56]

**Table 2:** Major absorption spectrum peaks in vivo of various AAPs.

Species	LH1 (nm)	Peripheral LH (nm)	Publication
*Acidiphilum rubrum*	864	-	[60]
*Acidisphaera rubrifaciens*	874	-	[61]
*Blastomonas aquatica*	866	-	[62]
*Chromatocurvus halotolerans*	877	-	[63]
*Congregibacter litoralis*	874	801, 850 (shoulder)	[64]
*Craurococcus roseus*	872	-	[65]
*Dinoroseobacter shibae*	868	804	[66]
*Erythroabcter dokdonensis*	862	835, 800	[67]
*Erythrobacter donghaensis*	867		[68]
*Erythromicrobium ramosum*	868	832, 798	[69]
*Gemmatimonas groenlandica*	863	-	[70]
*Gemmatimonas phototrophica*	866	819	[28]
*Hoeflea phototrophica*	865	805	[58]
*Limnohabitans planktonicus*	865	813, 799	[71]
*Luminiphilus syltensis*	871	-	[72]
*Paracraurococcus ruber*	856	-	[65]
*Photocaulis rubescens*	861	-	[73]
*Photocaulis sulfatitolerans*	860	-	[73]
*Polymorphobacter* sp. FW250	868	855 (shoulder), 800	[74]
*Pseudohaliea rubra*	871	804, 821	[72]
*Rhizobium* sp. BTAi 1	870	800	[75]
*Roseateles depolymerans*	873	-	[58]
*Roseibaca ekhonensis*	865	-	[76]
*Roseibacula alcaliphilum*	874	-	[77]
*Roseicitreum antarcticum*	872–874	-	[78]
*Roseicyclus elongatum*	879	802, 850 (shoulder)	[79]
*Roseicyclus mahoneyensis*	870	806	[80]
*Roseisalinus antarcticus*	870	-	[81]
*Roseivivax halodurans*	873	-	[82]
*Roseobacter litoralis*	868	806	[58]
*Roseococcus thiosulfatophilus*	856	-	[15]
*Roseomonas* sp. CK155	854	-	[14]
*Roseovarius tolerans*	878	-	[83]
*Sandaracinobacter sibiricus*	867	-	[84]
*Sandarakinorhabdus limnophila*	865	837, 800	[85]
*Vulcanimicrobium alpinum*	868	798	[30]

Atypical peripheral LH absorption peaks indicated in red (LH3), purple (LH4), and blue (LHh).

## Data Availability

The genomes used In Figure 1 and Figure 5 are available in GenBank under the assembly accession numbers provided in Supplementary Table S1.

## Supplementary Materials

The following supporting information can be downloaded at: https://www.mdpi.com/article/10.3390/microorganisms13112446/s1, Table S1: Genomes used in Figure 1 and Figure 5; Table S2: Abbreviations of species discussed.

## Abbreviations

The following abbreviations are used in this manuscript:

e^−^	Electron
BChl	Bacteriochlorophyll
AAP	Aerobic Anoxygenic Phototrophs
PGC	Photosynthetic Gene Cluster
PNSB	Purple Non-sulfur Bacteria
LH	Light-harvesting
RC	Reaction Center
BPhe	Bacteriopheophytin
Cyt	Cytochrome
HGT	Horizontal Gene Transfer
Chl	Chlorophyll
MAG	Metagenome-assembled Genome
