# Supplementary material for: Phenomenal Diversity of the Photosynthetic Apparatus Evolved in Aerobic Anoxygenic Phototrophs
Source: Microorganisms. 2025 Oct 25;13(11):2446. doi: 10.3390/microorganisms13112446 (PMC12654162; doi:10.3390/microorganisms13112446)
Supplement: Supplementary file 1 [file microorganisms-13-02446-s001.zip › microorganisms-3909018-supplementary.pdf]

Table S1. Genomes used in Figures 1 and 5.

Name	GenBank Assembly Accession Number
Acidiphilium rubrum	GCA_900156265.1
Acidisphaera rubrifaciens	GCA_000964365.1
Algirhabdus cladophorae	GCA_044772865.1
Aquincola tertiaricarbonis	GCA_023573145.1
Blastomonas ursincola	GCA_013607875.1
Brevundimonas aurifodinae	GCA_040195595.1
Chromatocurvus halotolerans	GCA_004340525.1
Congregibacter litoralis	GCA_000153125.2
Craurococcus roseus	GCA_039523795.1
Dinoroseobacter shibae	GCA_000018145.1
Elioraea tepida	GCA_019203965.1
Erythrobacter dokdonensis	GCA_002155305.1
Erythrobacter litoralis	GCA_001719165.1
Erythrobacter longus	GCA_000715015.1
Erythrobacter ramosus	GCA_014195675.1
Gemmatimonas groenlandica	GCA_013004105.1
Gemmatimonas phototrophica	GCA_000695095.2
Hoeflea phototrophica	GCA_000154705.2
Lichenicoccus roseus	GCA_005844085.1
Limnohabitans planktonicus	GCA_001270065.2
Luminiphilus syltensis	GCA_000158175.1
Paracraurococcus ruber	GCA_004353985.1
Photocaulis sulfatitolerans	GCA_027627705.1
Planktomarina temperata	GCA_000738435.1
Pseudohaliea rubra	GCA_000764025.1
Roseateles depolymerans	GCA_001483865.1
Roseibaca ekhonensis	GCA_900499075.1
Roseibium alexandrii	GCA_000158095.1
Roseicitreum antarcticum	GCA_014681765.1
Roseicyclus elongatus	GCA_000590925.1
Roseicyclus mahoneyensis	GCA_003148775.1
Roseinatronobacter thiooxidans	GCA_001870675.1
Roseisalinus antarcticus	GCA_900172355.1
Roseivivax halodurans	GCA_000521785.1
Roseobacter denitrificans	GCA_002983865.1
Roseobacter litoralis	GCA_000154785.2
Roseococcus thiosulfatophilus	GCA_017311575.1
Roseomonas fluvialis	GCA_022846615.1
Roseovarius tolerans	GCA_900109855.1
Rubrimonas cliftonensis	GCA_900107585.1
Rubrivivax pictus	GCA_005403045.1
Salinarimonas chemoclinalis	GCA_041514305.1
Sandarakinorhabdus limnophila	GCA_000420765.1
Sediminicoccus sp. KRV36	GCA_023243115.1
Sphingomonas sp. AAP5	GCA_004354345.1
Vulcanimicrobium alpinum	GCA_027923555.1
Yoonia vestfoldensis	GCA_000382265.1

Table S2. Abbreviations of species discussed.

Genus	Shortened name
Acidiphilium	*Ac.*
Allochromatium	*A.*
Blastomonas	*B.*
Brevundimonas	*Br.*
Cereibacter	*Cb.*
Chloroacidobacterium	*Ch.*
Chromatium	*Cr.*
Chromatocurvus	*C.*
Congergibacter	*Cg.*
Dinoroseobacter	*D.*
Erythrobacter	*E*
Erythromicrobium	*Er.*
Gemmatimonas	*G.*
Marichromatium	*Mc.*
Methylobacterium	*Mb.*
Methylorubrum	*M.*
Nereida	*N.*
Photocaulis	*Ph.*
Porphyrobacter	*P.*
Rhodoblastus	*Rhb.*
Rhodopseudomonas	*Rh.*
Roseateles	*Rt.*
Roseicyclus	*R.*
Roseobacter	*Rb.*
Roseococcus	*Rc.*
Sulfitobacter	*Sb.*
Vulcanimicrobium	*V.*
